# An Eastern County Hospital's Preparations

**Published:** 1917-02-10

**Authors:** 


### IN WARTIME.

### An Eastern County Hospital's Preparations.

By "EAST ANGLIA."

The excellent article in your issue of January 29 by Mr. E. W. Morris on the great explosion and its relation to the London Hospital must bring home to all hospital administrators the necessity for preparations to meet emergencies, more especially during this terrible war. The wide area covered by the Zeppelins during the past two years has induced each of many hospitals to make arrangements to cope with a call upon its resources should any bombs be dropped in its immediate vicinity. As this hospital is situated in that part of the country usually visited by hostile aircraft, the following air-raid emergency preparations in vogue here may be of interest to your readers. Happily, so far, our preparedness has not been put to the test, but should it be so at any time in the future, I trust this institution will not in any way be found wanting.

Our first warning of the fact that hostile aircraft are in the district is received by telephone from the local exchange. In order that there shall be no delay in the receipt of this warning, arrangements were made some time ago for a porter to be always in attendance in the porter's lodge, where the telephone is situated. In normal times we had only one night porter, but since the inauguration of air-raid warnings the work has been adjusted and a second man appointed, so that a porter shall always be in attendance to receive the telephone call.

Upon receipt of the warning the porter lights a number of hurricane lanterns, which are kept ready near at hand, and stands them in the hall. He then telephones to all wards and departments, where similar lanterns are lighted. By this time the electric lights are being gradually switched off at the electricity works, so that the need for the lanterns being first lighted is obvious. The gas is then turned off at the main, and should there be any need for light in either the operating theatres or surgery, emergency electric lights (the accumulators for which are charged periodically) are provided.

The greatest care is always exercised to ensure that no lights are visible from the outside of the building, so that with the enforced restricted lighting after an air-raid warning has been received there is practically no chance of the hospital lights being seen by the Zeppelin, which is the first precaution we are told to observe by the local authorities.

Incidentally, I may here refer to the very stringent lighting regulations in force. I have heard from several hospital secretaries that they have experienced trouble in complying with the regulations in this matter in their districts, but I have never yet seen a place where all lights have been subdued with such effect as in this city. I believe we have spent, since the commencement of the war, a sum approaching four figures in providing blinds, curtains, screens, etc., for this hospital, and we continue to spend money in this direction, for as the war progresses we find these blinds and screens require renewing. Although this is a matter occasioning much worry, we do not grumble, as we believe our immunity from an actual air-raid is due to the strong line on this point taken by our municipal authorities.

To return to the routine followed on air-raid nights. As the electric current is switched off, all members of the staff on duty, and most of the patients who may be awake, are aware of the fact that a raid is in progress. The lanterns which were first lighted and placed in the hall are distributed by one of the porters to various positions over the building, in order to give sufficient light for one to get around the hospital.

In the meantime, one of the house porters, all of whom have been informed of the position, is dispatched to arouse two of the oldest members of the hospital fire brigade, who live away from the institution. Our fire brigade consists of practically all the male employees of the hospital, and they have one hour's drill each week, the instructor being one of the chief officials of the local municipal fire brigade. The attendance of the men at these drills is practically compulsory, permission to be absent being only given in exceptional cases, and, in order that it shall not interfere with the work of the institution, the drills are held on a certain morning each week between six and seven o'clock. The men are paid for each attendance, and the two men who are brought from their homes on the receipt of the air-raid warning are paid for the number of hours they have to stay during the night. Owing to our geographical position special instruction is given at the fire drills for dealing with the effects of a direct attack by hostile aircraft—that is, the method of dealing with incendiary bombs, fumes, etc., and the removal of patients. In this way there are always in the building whilst a raid is in progress a certain number of men well versed in the method of dealing with fires and explosions. We have pails of water and sand in every part of the building, and the hydrants and hose are periodically inspected. Spare bedsteads are ready in the event of a big disaster in the district, and I think our arrangements for dealing with a large number of casualties would be found satisfactory.

Before closing this account of our emergency preparations I should like to refer to the matron of this hospital and the members of the nursing staff. The matron is called on receipt of the warning, and she remains on duty until word is received from the authorities that "normal conditions may be resumed." By the calm and collected way in which she visits the wards and departments during that time, speaking a cheery word to the nurses and patients, a spirit of confidence is created amongst the nurses on duty, and any sign of nerves experienced when the warning is first received is soon overcome. Our experiences on these air-raid nights are not by any means pleasant, and your readers will agree that a word of praise is due to the nursing staff for the calm way in which the work of the hospital has been maintained on very many nights when it was known that hostile aircraft were about, the falling of bombs has been heard, and Zeppelins were seen and heard passing overhead.

